# The multifaceted role of macrophages in homeostatic and injured skeletal muscle

**DOI:** 10.3389/fimmu.2023.1274816

**Published:** 2023-10-25

**Authors:** Xingyu Wang, Lan Zhou

**Affiliations:** Department of Neurology, Boston University Chobanian & Avedisian School of Medicine, Boston, MA, United States

**Keywords:** skeletal muscle, macrophage, homeostasis, injury repair, aging

## Abstract

Skeletal muscle is essential for body physical activity, energy metabolism, and temperature maintenance. It has excellent capabilities to maintain homeostasis and to regenerate after injury, which indispensably relies on muscle stem cells, satellite cells (MuSCs). The quiescence, activation, and differentiation of MuSCs are tightly regulated in homeostatic and regenerating muscles. Among the important regulators are intramuscular macrophages, which are functionally heterogeneous with different subtypes present in a spatiotemporal manner to regulate the balance of different MuSC statuses. During chronic injury and aging, intramuscular macrophages often undergo aberrant activation, which in turn disrupts muscle homeostasis and regenerative repair. Growing evidence suggests that the aberrant activation is mainly triggered by altered muscle microenvironment. The trained immunity that affects myeloid progenitors during hematopoiesis may also contribute. Aged immune system may contribute, in part, to the aging-related sarcopenia and compromised skeletal muscle injury repair. As macrophages are actively involved in the progression of many muscle diseases, manipulating their functional activation has become a promising therapeutic approach, which requires comprehensive knowledge of the cellular and molecular mechanisms underlying the diverse activation. To this end, we discuss here the current knowledge of multifaceted role of macrophages in skeletal muscle homeostasis, injury, and repair.

## Introduction

Skeletal muscle accounts for about 40% of human body mass. Besides being a crucial component of locomotor system, skeletal muscle contributes to basal energy metabolism and temperature maintenance. It also serves as an important secretory tissue, producing cytokines and other peptides (1, 2). Maintaining skeletal muscle homeostasis is critical to human health and wellbeing. Skeletal muscle homeostasis can be disrupted by many factors, such as acute injury, infection, genetic defect, and aging. Depending on the nature of the inciting factor, the outcome varies. Acute skeletal muscle injury, such as those caused by trauma or myotoxin exposure, can be repaired well unless the injury is repeated or too large. On the other hand, repeated acute injuries, large volumetric muscle loss, and chronic muscle injury caused by genetic defects cannot be completely repaired, which often leads to fibro-fatty tissue replacement in injured muscle. Muscle injury repair is a highly coordinated process, involving inflammation, myogenesis, revascularization, and remodeling of extracellular matrix (ECM) (3–5).

The capability of skeletal muscle to regenerate following injury relies on myogenic stem cells, muscle satellite cells (MuSCs), which largely stay quiescent in the homeostatic skeletal muscle (6). Depletion of MuSCs completely abolishes muscle regeneration (7). Injury-induced changes in tissue microenvironment activate MuSCs to regenerate muscle fibers. This process consists of a series of tightly-regulated proliferation and differentiation events, which have been reviewed in details by others (8–10). Briefly, quiescent MuSCs respond to extracellular signals released by injured muscle and become activated. Activated MuSCs proliferate and differentiate into myoblasts, which then fuse into multinucleated myotubes that further differentiate into muscle fibers (8–10). The quiescence, activation, and differentiation of MuSCs must be balanced to maintain stem cell pool in healthy muscle and to successfully regenerate in injured muscle. This balance is tightly regulated in a spatiotemporal manner by signals from the surrounding muscle microenvironment, which consist of extracellular matrix (ECM) and many different cell types including macrophages (11).

**Figure 1:**
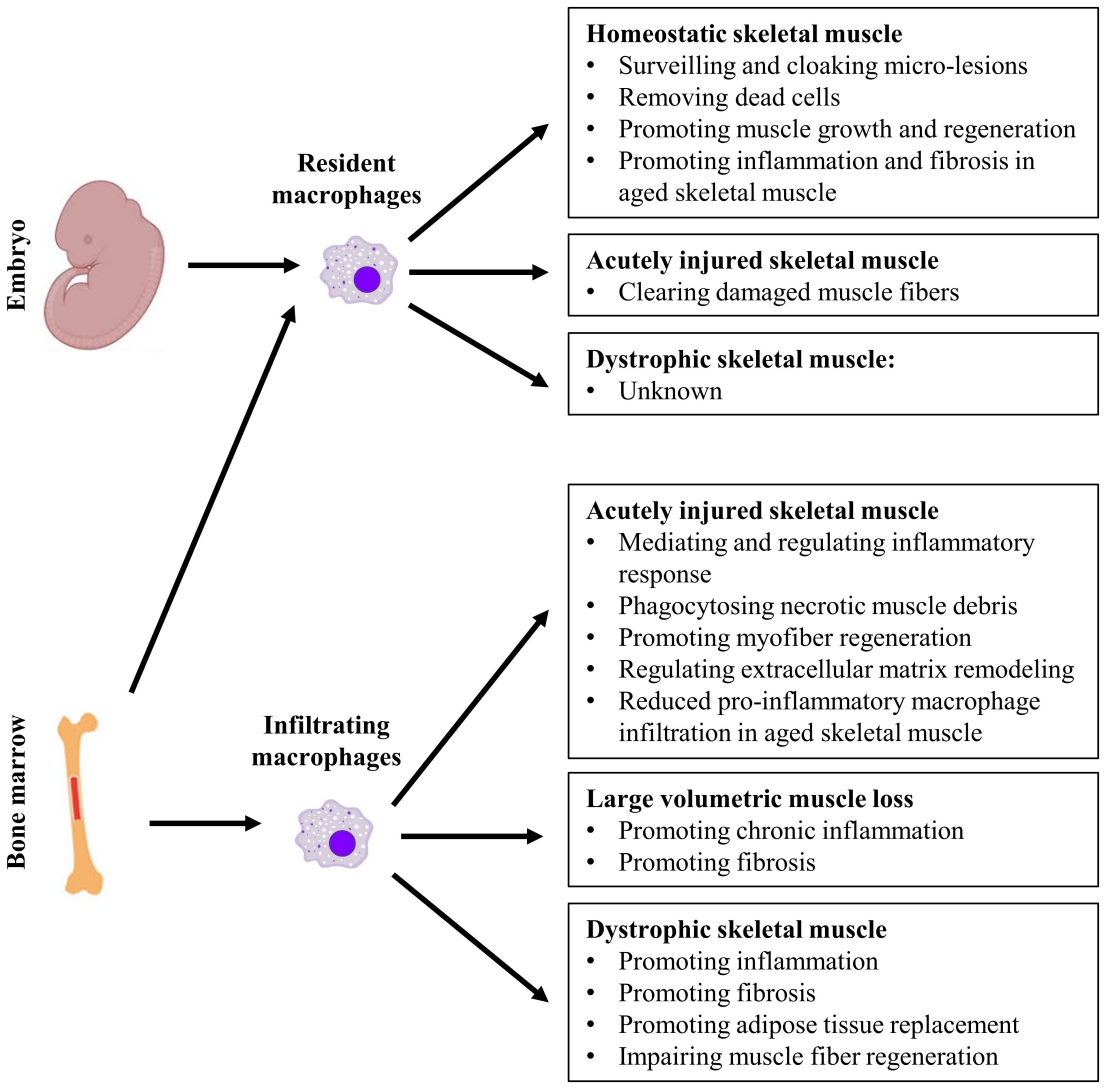
The multifaceted role of resident and infiltrating macrophages in homeostatic, acutely injured, and dystrophic skeletal muscles.

Macrophages predominate the inflammatory response to sterile muscle injury and contribute to the outcome of injury repair. They are functionally heterogeneous, arise from multiple origins, and actively participate in many biological processes including tissue homeostasis and injury repair (12–16). Resident macrophages maintain homeostasis of steady-state tissue via surveillance of local tissue microenvironment and response to changes. In a disease state, the functions of intramuscular macrophages can vary greatly depending on their origins and tissue microenvironment. They appear to be a double-edged sword. On the one hand, they could be essential to tissue regeneration, as seen during acute injury repair. On the other hand, they could contribute to tissue pathology, as seen during chronic inflammation (15–19). Macrophage has become a central focus of research in skeletal muscle injury and repair. In this review, we will discuss the multifaceted role of macrophages in skeletal muscle homeostasis, injury, and repair. The discussion will focus firstly on the essential roles of macrophages in maintaining homeostasis of steady state muscle and in regenerative repair of injured muscle. The pathological roles of macrophages during degenerative muscle injury repair will then be discussed. Lastly, we will discuss the roles of macrophages in aged muscle in the steady state and injury state (**Figure 1**).

## The origins and functions of resident macrophages in homeostatic skeletal muscle

Tissue macrophages are classified into resident macrophages and infiltrating inflammatory macrophages. Resident macrophages are usually established during embryogenesis and are present in all adult tissues including skeletal muscle (20). Adult tissue resident macrophages originate from both embryonic and adult hematopoiesis. During embryonic hematopoiesis, tissue macrophages can be derived firstly from primitive yolk sac macrophages and secondly from fetal liver monocytes (fetal monocytes) (14, 21–28). Fetal monocytes gradually outcompete yolk sac macrophages to seed all embryonic tissues except for brain (27). Embryonic tissue resident macrophages can persist into adulthood through self-renewal. Adult hematopoiesis is originated from bone marrow hematopoietic stem cells (HSCs), which give rise to blood monocytes (adult monocytes) after birth. Adult monocytes contribute to the replenishment of resident macrophages in many tissues (29–33), including skeletal muscle (20). Different from tissue resident macrophages, infiltrating macrophages are exclusively derived from blood monocytes originated from bone marrow HSCs.

Combining single cell-based RNA sequencing (scRNAseq) and lineage-tracing technique, a recent study identified three common resident macrophage subtypes across different murine tissues, including skeletal muscle, and these functional subtypes correlate with the macrophage origins (34). The identification was based on the expression of four genes: *Timd4*, *Lyve1*, *Folr2*, and *Ccr2*. The first subtype expresses *Timd4* and/or *Lyve1* and/or *Folr2* (*TLF* ^+^), and they are self-renewal macrophages originated from yolk sac and fetal monocyte precursors. The second subtype is *TLF^-^Ccr2* ^+^ which can be entirely replaced by blood monocytes. The third subtype is *TLF^-^Ccr2* ^-^ which expresses a high level of MHCII molecules and receives a moderate contribution from adult monocytes (34). Likewise, another study using scRNAseq analysis of human macrophages also showed that the *Lyve1^+^* macrophages overlap strongly with fetal liver macrophages, suggesting an embryonic origin of this subtype, while the *Lyve1* ^-^ macrophages expressed monocytic signature genes, suggesting their monocytic origin (35). Similar resident macrophage subtypes have also been identified in skeletal muscle by other studies (20, 36). One study, combining scRNAseq and lineage-tracing, showed that adult skeletal muscle resident macrophages mostly arise from fetal monocytes and adult bone marrow HSCs, with a small percentage from primitive macrophages (20). In this study, the authors identified two macrophage subtypes: the CCR2^+^MHCII^hi^Lyve1^low^ macrophages which are completely derived from blood monocytes, and the CCR2^-^MHCII^low^Lyve1^hi^ macrophages which mainly arise from embryonic origins (20). In addition, *Folr2* expression is higher in CCR2^-^ macrophages than in CCR2^+^ macrophages (20). Another study, using parabiosis and single-cell transcriptome analysis, identified similar muscle resident macrophage subtypes: self-renewal TIM4^+^Lyve1^+^CCR2^-^ cells and blood replenished TIM4^-^Lyve1^-^CCR2^+^ cells (36). Together, these findings indicate that while the *TLF* expression primarily marks the embryo-derived resident macrophages, the CCR2 and MHCII expression preferentially marks the adult monocytes-derived resident macrophages in skeletal muscle. These differentially expressed markers provide useful tools for studying muscle resident macrophage subtypes of different origins. We summarize the origins, life cycles, and markers of different subpopulations of resident macrophages as well as infiltrating macrophages in **Table 1**.

**Table 1:** Origins and markers of intramuscular macrophages.

Macrophage type	Subpopulation	Origins	Life cycle	Transcriptional signature
Resident Macrophage	TLF^+^CCR2^-^	embryonic hematopoiesis	proliferative self renewal	Timd4^+^ and/or Lyve1^+^ and/or Forl2^+^, Ccr2^-^, MHCII^low^, Ly6C^lo^
	TLF^-^CCR2^+^	adult blood monocytes	proliferative self renewal and replenishment by blood monocytes	Timd4^-^ and/or Lyve1^-^ and/or Forl2^-^, Ccr2^+^, MHCII^hi^, Ly6C^lo^
	TLF^-^CCR2^-^	embryonic hematopoiesis and adult blood monocytes	proliferative self renewal and replenishment by blood monocytes	Timd4^-^ and/or Lyve1^-^ and/or Forl2^-^, Ccr2^-^, MHCII ^hi^, Ly6C^lo^
Infiltrating Macrophage	CCR2^+^Ly6C^hi^	adult blood monocytes	blood moncytes recruitment	Timd4^-^ and/or Lyve1^-^ and/or Forl2^-^, Ccr2^+^, MHCII^low^

Skeletal muscle resident macrophages reside in interstitial tissue as CD45^+^F4/80^+^CD64^+^ Ly6C^lo^ cells, and they express muscle-specific transcription factors and display muscle-specific functions (20). Compared to the resident macrophages in other tissues, resident macrophages in skeletal muscle display a distinct transcriptome profile, which is characterized by enriched expression of genes involved in maintenance of muscle homeostasis and promotion of muscle growth and regeneration (20). Skeletal muscle resident macrophages also exhibit muscle-type specificity, as demonstrated by a much higher expression level of stress response genes by respiratory muscle resident macrophages than by limb muscle resident macrophages (20). An independent study provided additional evidence to support the homeostatic function of skeletal muscle resident macrophages (37). In multiple tissues including skeletal muscle, resident macrophages were shown to rapidly cloak tissue microlesions to prevent neutrophil chemotaxis and subsequent inflammatory tissue damage (37). This functional property of resident macrophages prevented complete death of myofibers from microlesions and maintained structural integrity of muscle in the steady state (37). Maintaining tissue homeostasis appears a ubiquitous function of resident macrophages across different tissues thanks to their ability to phagocytose dead cells and debris and to suppress immune activation (38). In addition, the transcriptome study suggests that muscle resident macrophages of different origins are specialized to exert different functions. While the CCR2^+^MHCII^hi^Lyve1^low^ macrophages, which mainly arise from HSCs, are more antigen presenting cells, the CCR2^-^MHCII^low^Lyve1^hi^ macrophages, which arise from both HSCs and non-HSCs embryonic progenitors, are more active phagocytes (20). Notably, the CCR2^-^MHCII^low^Lyve1^hi^ subtype accounts for almost 70% of the total muscle resident macrophages (20), and the percentage is similarly to that of the TLF^+^ resident macrophages in heart but much higher than that of the TLF^+^ macrophages in other tissues (34). Cardiac tissue shares similarity to skeletal muscle. Cardiac resident macrophages are more studied and better understood than skeletal muscle resident macrophages. It was reported that LYVE1^+^ (TLF^+^) cardiac resident macrophages are localized to the areas near blood vessels (39), which might be due to the ability of LYVE1 to bind hyaluronic acid on smooth muscle cells in blood vessel walls (40). Considering both heart and skeletal muscles are highly vascularized organs, proximity to vasculature may account for a high percentage of TLF^+^ resident macrophages in these two tissues. Cardiac TLF^+^ resident macrophages are also reported to be more active in phagocytosing dead cells and tissue debris (30, 41), like the counterpart in skeletal muscle. They are also required for appropriate vascular patterning and lymphatic development (42, 43). It would interesting to explore whether muscle TLF^+^ resident macrophages have similar functions. On the other hand, LVVE1^lo^MHCII^hi^ cardiac resident macrophages reside near nerves (39), with their functional significance not yet known in homeostatic heart. The localization and functions of skeletal muscle resident macrophage subtypes need to be further studied in the future.

## The essential roles of macrophages in skeletal muscle regeneration following acute injury

A number of animal models have been used to study the roles of macrophages in acute skeletal muscle injury repair. In these models, the injury is induced by mechanical damage, contusion, freeze, myotoxin or heavy metal salt injection, or ischemia. The technical merits of different acute injury models are reviewed by Baghdadi et al. (44). Studies based on different models have revealed a similar repair process with small differences. Except for repeated injuries (45) and a large volume of muscle loss (46, 47), acute skeletal muscle injury usually repairs within 2 to 3 weeks without significant residual defects. Although the regeneration of damaged myofibers relies on the activation and differentiation of MuSCs (8–10), an adequate inflammatory response, triggered by injury and predominated by neutrophil and macrophage infiltration, is required for the complete muscle regeneration (3, 48–50). Both muscle resident macrophages and infiltrating macrophages play essential roles in acute skeletal muscle injury repair.

### Resident macrophages are required for acute skeletal muscle injury repair

Studying the role of resident macrophages in muscle injury and repair has been long hampered due to the lack of specific markers to separate resident macrophages from infiltrating macrophages and the lack of effective approach to selectively target resident macrophages. The recent identification of TIM4 and Lyve1 as markers for self-renewal muscle resident macrophages helps facilitate such studies (36). By using a CSF1R inhibition and withdrawal strategy, TIM4^+^Lyve1^+^ resident macrophages can be selectively and temporally depleted from steady-state skeletal muscle (36). Using this model, one study showed that depletion of TIM4^+^Lyve1^+^ resident macrophages at the early stage of acute muscle injury repair resulted in prolonged accumulation of necrotic fibers and impaired muscle regeneration. Therefore, this subtype of self-renewal resident macrophages is required for efficient clearance of damaged fibers to promote muscle regeneration (36). Single-cell transcriptome analysis showed enriched expression of genes involved in phagocytosis and inflammatory cell chemotaxis in the TIM4^+^Lyve1^+^ resident macrophages after muscle injury, suggesting that this subtype may also play a role in initiating inflammation (36). However, the recruitment of neutrophils and inflammatory monocytes did not change after the depletion of TIM4^+^Lyve1^+^ resident macrophages, suggesting that other resident cells, including TIM4^-^Lyve1^-^ resident macrophages, may compensate for this role (36). The contribution of resident macrophages to acute skeletal muscle injury repair remains largely unknown and needs to be further explored, particularly with advanced techniques such as lineage tracing and single-cell based analysis. Findings from research in cardiac resident macrophages may provide useful hints, given the similarity between these two types of tissues. Following myocardial injury, transcriptional programs of cardiac resident macrophages are induced to primarily promote phagocytosis and efferocytosis (51, 52), suggesting that cardiac resident macrophages are also required for efficient clearance of damaged tissue debris, similar to their counterpart in skeletal muscle. Self-renewing CCR2^-^ resident cardiac macrophages resident cardiac macrophages also limit adverse remodeling (51, 53). While the CCR2^+^ subset promotes recruitment of neutrophils (54) and monocytes (53), the CCR2^-^ subset inhibits monocytes recruitment (53). Whether the different subtypes of skeletal muscle resident macrophages perform similar roles following acute injury is yet to be determined.

### Infiltrating macrophages, differentiated from circulation-derived inflammatory monocytes, undergo phenotypic switch during acute skeletal muscle injury repair

In acutely injured skeletal muscle, infiltrating macrophages are differentiated from HSC-derived blood monocytes (55, 56), which consist of two different subsets in mice: Ly6C^hi^CCR2^+^CX3CR1^lo^ and Ly6C^lo^CCR2^-^CX3CR1^hi^ monocytes (57). Injured skeletal muscle recruits Ly6C^hi^CCR2^+^CX3CR1^lo^ (57) but not Ly6C^lo^CCR2^-^CX3CR1^hi^ (58) monocytes through CC chemokine receptor 2 (CCR2) chemotaxis signaling (56, 59–62). Ly6C^hi^CCR2^+^CX3CR1^lo^ monocytes then differentiate into Ly6C^hi^ inflammatory macrophages. In addition to blood circulation, Ly6C^hi^ monocytes can also be deployed from spleen reservoir into inflamed tissues, including injured skeletal muscle (63, 64). After phagocytosing necrotic muscle debris, intramuscular Ly6C^hi^ macrophages can switch into Ly6C^lo^ macrophages (56, 65–69). This Ly6C^hi^-to-Ly6C^lo^ switch, rather than the pre-existing resident macrophages, makes the major contribution to the Ly6C^lo^ macrophage accumulation in injured muscle (56, 69, 70). The CD14^hi^CD16^low^ and CD14^low^CD16^hi^ monocytes in humans are the counterparts of Ly6C^hi^ and Ly6C^lo^ monocytes in mice, respectively (71).

The Ly6C^hi^-to-Ly6C^lo^ switch during skeletal muscle injury repair suggests major functional changes of macrophages accompanying the injury repair process. Ly6C^hi^ and Ly6C^lo^ macrophages have once been considered to correlate with the bipolar classification of M1 (pro-inflammatory) and M2 (anti-inflammatory, pro-regenerative, and/or pro-fibrotic) macrophages, respectively (72, 73). Accompanying the Ly6C^hi^-to-Ly6C^lo^ switch, infiltrating macrophages indeed undergo pro- to anti-inflammatory functional phenotype switch, as reflected by the switches of pro- to anti-inflammatory gene expression (15, 19, 56, 65, 70, 74–77) and pro- to anti-inflammatory lipid mediator production (78, 79). However, *in vivo* macrophage studies indicate that this simplified bipolar paradigm of macrophage activation does not fit complex *in vivo* scenario (72, 80, 81). Macrophages are highly plastic cells and can rapidly switch their functional status in response to the changes of tissue microenvironment, leading to diverse macrophage subtypes. In a study using single-cell transcriptome analysis of acutely injured muscle, several macrophage subtypes were identified sequentially post injury: 1) a more pro-inflammatory subtype at day 2; 2) an *Il7r* ^+^ subtype from day 3.5 to day 5; 3) a complement genes-enriched subtype from day 3.5 to day 10; 4) a subtype with enriched expression of genes of antigen presentation from day 5 to 10. Different macrophage subtypes coexist at multiple time points, with the fraction of each cluster changing over time (82). Another study found that the expression pattern of M1 and M2 genes is similar in Ly6C^hi^ and Ly6C^lo^ macrophages at day 3 post injury, but different between Ly6C^hi^ macrophages at day 1 and Ly6C^hi^ macrophages at day 3 post injury (70). Therefore, the changes of infiltrating macrophage phenotype appears to be driven by the changes in the tissue microenvironment rather than the Ly6C status (70, 82, 83).

### Infiltrating macrophages play critical roles in regulating acute skeletal muscle injury repair

Infiltrating macrophages are required for the complete repair of acutely injured muscle, during which macrophages of different activation status actively regulate inflammation, myogenesis, and ECM remodeling in a spatiotemporal manner. Disruption of macrophage infiltration or their spatiotemporal phenotype switch results in impaired muscle regeneration which is often accompanied by muscle fibrosis (56, 59–62, 76, 84, 85). These findings also suggest that the pre-existing resident macrophages cannot compensate the roles of infiltrating macrophages to adequately support acute muscle injury repair.

Infiltrating macrophages mediate and regulate the inflammatory response to acute muscle injury. They produce a high level of pro-inflammatory mediators at the early stage for inflammation propagation, while produce a high level of anti-inflammatory mediators at the later stage for inflammation resolution. In addition, the early infiltrated Ly6C^hi^ macrophages are also required to clear damaged muscle debris, as blocking macrophage infiltration via CCR2 deficiency delays the clearance of necrotic muscle fibers (61). Phagocytosis of damaged tissue debris appears to drive the pro- to anti-inflammatory phenotype switch of macrophages (56, 65–69).

Infiltrating macrophages promote muscle regeneration by regulating the core process of myogenic cell activation, proliferation, differentiation, and fusion after injury. Different subtypes of macrophages appear to exert different functions. Pro-inflammatory macrophages promote myogenic cell activation and proliferation, while anti-inflammatory macrophages promote myogenic cell differentiation and fusion (56, 86–88). This is further supported by histopathological analysis of injured skeletal muscle, which shows co-localization of pro-inflammatory macrophages with proliferating satellite cells and co-localization of anti-inflammatory macrophages with differentiated myoblasts (77). Pro-inflammatory macrophages produce a high level of soluble factors known to stimulate the activation and proliferation of MuSCs, including fibronectin (89), IL-6 (90), TNF-α (91), PGE2 (92), and A Disintegrin-Like and Metalloproteinase with Thrombospondin Type 1 Motif (ADAMTS1) (93). On the other hand, anti-inflammatory macrophages produce factors that can stimulate myoblast differentiation and myofiber growth, including IL-4 (94), IGF-1 (61, 95, 96), and GDF-3 (97). The increased glutamine synthesis that accompanies the macrophage phenotype switch during acute injury repair has also been shown to boost satellite cell activation and muscle regeneration (98). Therefore, the spatiotemporal presence of macrophages of different phenotypes appears important to the proper muscle regeneration after injury. This hypothesis is further supported by the findings that targeting signaling molecules that critically involved in macrophage phenotype switch, including IGF-1 (96), Meteorin-like (Metrnl) (99), AMP-activated protein kinase-1 (AMPKα1) (65, 100), Nuclear Factor IX (Nfix) (68), CCAAT/enhancer binding protein-β (C/EBPβ) (77), and peroxisome proliferator-activated receptor-γ (PPARγ) (97), impairs muscle regeneration.

Infiltrating macrophages regulate ECM remodeling, a temporary ECM deposition and degradation process that provides an essential structural support for myogenic cell activation and differentiation during acute muscle injury repair. Besides structural support, some ECM components, such as collagen 6a (Col6a) (101) and fibronectin (89), can activate MuSCs. The primary cells that produce ECM are fibro/adipogenic progenitors (FAPs), which also support myogenic cell activation and differentiation to facilitate muscle regeneration (7, 102–104). However, impaired regenerative process can lead to aberrant FAP activation, which results in fibro-fatty tissue replacement and failure to support MuSC activation (104). Macrophages have been shown to regulate the accumulation and activation of FAPs in injured skeletal muscle. The pro-inflammatory macrophages can induce FAPs apoptosis by secreting TNF-α (105) to limit excessive FAP accumulation. On the other hand, the anti-inflammatory macrophages can enhance proliferation of fibrogenic cells through increased production pro-fibrotic factors such as TGF-β1 (106). Depleting macrophages or blocking macrophage recruitment results in not only poor muscle regeneration but also muscle fibrosis (60, 105), further supporting the importance of infiltrating macrophages in regulating FAP activity and ECM remodeling.

In summary, both resident macrophages and infiltrating macrophages are required for the complete repair of acutely injured skeletal muscle. While both resident and infiltrating macrophages clear dead cells and tissue debris, infiltrating macrophages of different activation status also actively regulate the activation, proliferation, differentiation, and growth of myogenic cells in a spatiotemporal for proper muscle regeneration (**Figure 1**).

## Microenvironment in injured muscle aberrantly activates macrophages to drive fibrosis in degenerative volumetric muscle loss

Acute skeletal muscle injury usually regenerates well. However, when a critical volume of muscle is lost due to surgery or trauma, a condition known as degenerative volumetric muscle loss (VML), muscle regeneration is compromised, along with prolonged inflammatory cell infiltration and muscle fibrosis (46, 47, 107–112). VML results in pronounced disabilities ranging from atrophy and dysfunction of muscle to aggressive development of osteoarthritis (112). Comparing to the regenerative acute skeletal muscle injury, degenerative VML causes prolonged macrophage infiltration that can persist for months (108). More importantly, the macrophage phenotypes in degenerative VML appear different from those in regenerative muscle injury (110, 113).

In a well-designed study, Anderson et al. determined the threshold of critical size of muscle loss that leads to degenerative injury repair following VML (114). In mouse quadriceps, full-thickness defect of 3 mm, corresponding to 15% of total mass, is the critical threshold of un-recoverable muscle loss. Twenty-eight days post injury, quadriceps with 3-mm but not 2-mm injury showed incomplete bridging of myofibers through the defect site, persistent inflammation, fibrosis, and increased central nucleated myofibers (114). This study established an excellent mouse model for exploring cellular and molecular mechanisms underlying the pathological outcomes following VML. Single cell-based analysis exploiting this model revealed significant differences in inflammatory cell infiltration and function in the degenerative VML injury comparing to the regenerative VML injury (109, 113). While the abundance of macrophages was significantly lower in the 3-mm than in the 2-mm injured muscles, the abundance of all the other inflammatory cell types identified, including monocytes and neutrophils, was persistently increased (109). Another study of VML injury in tibias anterior muscle analyzed the spatiotemporal function of macrophages (113). By combining spatial transcriptomics and scRNAseq techniques, the authors identified an aberrantly activated scar-associated macrophage (SAM) subtype, which has been reported in other diseased tissues, including cardiac muscle (115, 116), liver (117), and lung (118). SAMs feature the expression of both pro-inflammatory and anti-inflammatory genes, as well as pro-fibrotic genes including *Trem2* and *Spp1*. In the defect site of day 7-injured muscle, SAMs co-localize with mesenchymal-derived cells (MDCs), creating an inflammatory and pro-fibrotic milieu (113). The pro-fibrotic milieu persists 14 days post injury and leads to excessive collagen deposition (113). The study established a role for the aberrantly activated, pro-fibrotic biased macrophages in driving the pathological outcome of VML. By generating 2-mm and 3-mm VML injuries in the left and right quadriceps muscles of the same mice, respectively, leaving the size of the defect the only difference between the two injuries, Larouche et al. demonstrates that the aberrant activation of macrophages following critical VML is induced purely by the altered tissue microenvironment, which disrupts the spatiotemporal macrophage phenotype switch that normally observed during regenerative muscle injury repair (109). Therefore, improving tissue microenvironment by analyzing and targeting its molecular and cellular components may help promote injury repair.

## Heterogeneous and pathogenic activation of macrophages in muscular dystrophy is likely induced by a combination of tissue microenvironment and trained immunity

Muscular dystrophies consist of a heterogeneous group of genetic diseases characterized by progressive muscle atrophy and weakness (119). In severe cases, such as Duchene Muscular Dystrophy (DMD), patients die prematurely from respiratory and cardiac muscle weakness (119, 120). Many muscular dystrophies are caused by mutations in the genes encoding proteins of dystrophin-glycoprotein complex (DGC) or proteins required for the proper assembly of DGC, which in turn causes fragile sarcolemma and leads to myofiber necrosis (121). These genetic defects lead to a continuous cycle of muscle degeneration and regeneration, resulting in chronic inflammation and fibro-fatty tissue replacement (122). The most studied muscular dystrophy is DMD, which is caused by the defective dystrophin gene on the X chromosome (120).

### The pleiotropic roles of macrophages in dystrophic muscles of mdx mice

Most of the DMD studies are conducted in animal models, among which the most commonly used is *mdx* mice. *Mdx* mice display a milder phenotype compared to DMD patients (123–126). Muscle inflammation starts about 3 weeks of age, persists into 2-3 months of age, and then gradually subsides in limb muscles but not diaphragm (123–127). Correspondingly, progressive fibrosis takes place in diaphragm but not limb muscles. Along with the fibrosis development in diaphragm, respiratory function of *mdx* mice is impaired, which resembles human DMD patients (125, 127–129). Similar to acute injury, inflammation in *mdx* muscles is also predominated by macrophage infiltration (127). Recruitment of Ly6C^hi^ monocytes by *mdx* muscles also depends on CCR2 (130, 131), and intramuscular Ly6C^hi^-to-Ly6C^lo^ macrophage switch also occurs (130, 131). As comprehensive human study of DMD is lacking, we focus our discussion on the mouse model studies.

Macrophages in *mdx* leg muscles display diverse phenotypes depending on age: they appear more pro-inflammatory at 4 weeks while more pro-regenerative at 12 weeks (132). Depletion of macrophages at 4 weeks of age reduces leg muscle necrosis (133), which suggests reduced inflammation-induced muscle damage. A recent study exploiting scRNAseq analysis revealed that macrophages are highly heterogeneous in the leg muscle of 8-week-old *mdx* mice (134). The authors identified multiple macrophage subtypes with distinct transcriptomes, including two resident macrophage-like subtypes resembling resident macrophages in *WT* muscle, a pro-inflammatory subtype, an *Spp1*-expressing subtype reportedly to be pro-fibrotic in other tissues (135, 136), and several low-fraction subtypes (134). Importantly, the abundance of the pro-inflammatory subtype and the *Spp1*-expressing subtype correlated with the disease severity (134), indicating the pathogenicity of these macrophage subtypes. An independent study also indicates that Ly6C^hi^ macrophages contribute to the fibrosis of *mdx* leg muscles at 8 to 10 weeks of age by producing latent TGF-β1 (137). Consistently, multiple other studies also suggest that macrophages are generally pathogenic in *mdx* muscles, as reducing macrophage infiltration decreases muscle damage and fibrosis while improves muscle function before 3 months of age (64, 130, 131, 138). Contrarily, there is evidence suggesting that anti-inflammatory macrophages may be beneficial to *mdx* (126, 127). Depleting macrophages from *mdx* limb muscles at 10 to 12 weeks of age compromised myogenic cell proliferation and differentiation, decreased myofiber formation, and increased fibro-fatty tissue deposition (139). Studies concerning macrophage-derived factors, including TNF-α, IL-1β, IL-6, iNOS, IL-10, IGF-1, TGF-β, and osteopontin (encoded by *Spp1*), provide additional evidence of the pleiotropic roles of macrophages in *mdx* muscles, as these factors are critically involved in inflammation, regeneration, and fibrosis of the dystrophic muscles (48, 105, 127, 132, 140–142).

Despite the fact that diaphragm but not limb muscle displays persistent inflammation and progressive fibrosis in *mdx* mice, the function of macrophages in the *mdx* diaphragm is poorly understood compared to that in the *mdx* limb muscles. One study showed that in 14-week-old *mdx* mice, Ly6C^lo^ macrophages from diaphragm are more pro-inflammatory and pro-fibrotic than those from quadriceps (106). However, the knowledge is far from comprehensive. Future macrophage studies, particularly at a single-cell level, are required for this human DMD-resembling muscle type to provide further insight into the pathogenic roles of macrophages in muscular dystrophy.

### Heterogeneous and pathogenic activation of macrophages in mdx is likely induced by a combination of tissue microenvironment and trained immunity

Macrophages are highly plastic, responding and adapting to tissue microenvironment changes. During chronic inflammation, Ly6C^hi^ monocytes/macrophages continuously infiltrate and switch to Ly6C^lo^ macrophages in response to the constant damage of skeletal muscle. This creates a continuously altered microenvironment with mixed stimuli of both pro- and anti-inflammatory natures, disrupting the spatiotemporal presence of pro- and anti-inflammatory macrophages. Consequently, muscle regeneration is compromised. A simplified demonstration of this scenario is from a study of repeated acute skeletal muscle injury, in which muscle injury was induced twice at an interval of 4 or 10 days (45). The repeated injuries cause simultaneous presence of both pro- and anti-inflammatory macrophages in the injured muscle, as well as persistent inflammation, fibrosis, and impaired muscle regeneration (45). The altered microenvironment caused by continuous injuries in dystrophic muscle may account for the highly heterogeneous and pathogenic macrophage activation in *mdx* muscle (134).

Besides the asynchronous regenerative microenvironment in dystrophic muscle, a recent study revealed “trained immunity” as another important mechanism that drives the pathogenic monocyte/macrophage activation during bone marrow hematopoiesis in *mdx* mice (143). Trained immunity originally refers to a status of innate immune hyper-responsiveness induced by the exposure to infectious agents (144, 145). Macrophages of this status persist long after the clearance of the initial inducers, representing a type of innate immune memory. Importantly, this innate immune memory is not antigen specific and thus can promote exaggerated cytokine response to unrelated pathological challenges (144). Mechanistic studies have linked trained immunity to histone modifications (146–148) and changes in cellular metabolism (149). In addition to infections, damage-associated molecular patterns (DAMPs), released by necrotic cells and ECM breakdown during tissue injury, can also induce trained immunity (150, 151). DAMPs are recognized by pattern recognition receptors (PRRs) to trigger activation of intracellular signaling pathways that mediate the expression of inflammatory genes (152–156). PRRs, such as toll-like receptors (TLRs), have been shown to regulate trained immunity in monocytes/macrophages (143, 157). Trained immunity can be induced at the level of myeloid progenitors within bone marrow (143, 158–160), allowing its potential role in the progression of a chronic disease. Compared to wild-type (*WT*) mice, bone marrow-derived macrophages (BMDMs) from *mdx* mice showed a significantly increased basal expression of both pro- and anti-inflammatory genes. *Mdx* BMDMs also responded in an exaggerated fashion to heterologous inflammatory stimuli (143). This phenotypic reprogramming of BMDMs can be induced by DAMPs prepared from crushed skeletal muscle extract, and can be sustained after adoptive *mdx*-to-*WT* bone marrow transfer. TLR4 was shown to regulate the altered phenotype of *mdx* BMDMs through epigenetic reprogramming (143). These findings suggest that the aberrant and pathogenic macrophage activation in chronic dystrophic muscle may be contributed by the trained immunity in addition to the altered local muscle environment.

## Macrophages in skeletal muscle aging

With aging, skeletal muscle suffers loss of muscle mass and concurrent accretion of fat and connective tissues, a clinical syndrome called “*sarcopenia*” (161, 162). Aging also impairs the capability of skeletal muscle to regenerate after injury, leading to, similar to *sacopenia*, the decrease in the number and size of myofibers and the deposition of adipose and fibrous tissues (163–166). It is not clear whether these two age-related defects of skeletal muscle are interconnected. They both lead to declined muscle function and compromised health condition in the elderly. Understanding the mechanisms underlying these defects is therefore critical to the development of clinical strategies to improve the function of aged skeletal muscle.

### Aged immune system drives the aging of skeletal muscle

Aging impacts all tissues including immune system. Aged immune system loses its ability to mount an efficient immune response to infection or injury (167, 168). Immune cells, primarily the innate immune cells like macrophages, persistently infiltrate homeostatic tissues at a low level, and the level of several pro-inflammatory cytokines and chemokines, such as TNF-α and CCL2, increases in both tissues and circulation with aging (168, 169). These alterations lead to a hypothesis that aged immune system plays an important role in driving the aging of other tissues. This hypothesis gained a strong support from the work by Yousefzadeh et al. (170). The authors created a mouse model in which the increased burden of endogenous DNA damage was specifically induced in hematopoietic cells to cause pre-mature senescence in the immune system only. Interestingly, pre-mature senescence was also observed in non-lymphoid organs in this model, suggesting that aged immune cells can promote systemic aging. Transplantation of splenocytes from the mutant mice or aged wild-type mice into young mice induced senescence in the recipients, whereas transplantation of splenocytes from young mice into old mice suppressed senescence and tissue damage in aged recipients. In particular, the authors also showed impaired skeletal muscle regeneration following injury in the mutant mice as compared to the age-matched wild-type mice. Therefore, aged immune system plays a causal role in the aging of other tissues including skeletal muscle (170). This concept is further supported by another study showing that transplantation of young bone marrow cells into old recipients prevented *sarcopenia*, whereas transplantation of old bone marrow cells into young recipients reduced satellite cell number and promoted satellite cells to switch towards a fibrogenic phenotype (171).

### Altered phenotype of resident macrophages in aged skeletal muscle may contribute to sarcopenia

As macrophage is the predominant immune cell type in both homeostatic and regenerating skeletal muscle, they could potentially contribute to the defects of aged skeletal muscle. Aging results in a plethora of phenotypic and functional changes in macrophages, including but not limited to the increased production of pro-inflammatory mediators such as TNF-α and IL-1β and decreased capacity of phagocytosis (170, 172). In aged mouse skeletal muscle, a recent study combining scRNAseq and flow cytometry analysis revealed significant changes in macrophage functional phenotype (173). The authors showed increased fraction of Lyve1^-^ macrophages whereas decreased fraction of Lyve1^+^ macrophages in old muscle compared to young muscle, despite that the total macrophage number did not change (173). This is likely due to the continuous replenishment of embryo-derived muscle resident macrophages by blood monocytes (20). Accordingly, the expression levels of pro-inflammatory and senescence-related markers is increased in old skeletal muscle macrophages (173), suggesting an overall phenotypic shift towards a pro-inflammatory state with aging. Concurrently, the expression of *Spp1* gene, which was associated with pro-fibrotic function (135, 136), was also increased in the old skeletal muscle macrophages (173). Although the authors identified multiple macrophage sub-clusters, the relative change in the fraction of these clusters between old and young muscles was not shown (173). It is thus unclear whether the phenotypic shift is due to a selective expansion of specific macrophage subtypes or a general increase in gene expression. Nevertheless, the pro-inflammatory phenotype of aged skeletal muscle macrophages may contribute to the aging of skeletal muscle, as one study showed that myeloid cell-derived TNFα promoted *sarcopenia* (174). In addition, CD68^+^CD206^+^ macrophages have been shown to co-localize with intramuscular adipose tissue in old human and mouse skeletal muscle (175), suggesting a potential contribution of macrophages to the aging-related adipogenic replacement of myofibers.

### Aging-related changes in infiltrating macrophages may contribute to the compromised injury repair in aged muscle

Growing evidence supports the notion that aging compromises the ability of skeletal muscle to regenerate following injury (163–166). As macrophages are essential for supporting muscle regeneration, the altered macrophage function may account, in part, for the aging-related regeneration defect. Indeed, decreased infiltration of pro-inflammatory macrophages in old muscles compared to young ones was reported in mouse disuse atrophy (176) and human eccentric contraction injury (177). The delivery of pro-inflammatory macrophages to the muscle that underwent disuse atrophy promoted the recovery of muscle strength in aged mice (178). Thus, the aging-related decrease in pro-inflammatory macrophage infiltration likely contributes to the compromised injury repair of aged muscle. The functional activation of infiltrating macrophages after injury also appears altered in aged muscle. One study showed that the expression of IFN‐γ responsive genes by macrophages was down‐regulated in the regenerating muscle of aged mice after injury, and IFN‐γ deficiency impaired muscle regeneration (179). By scRNAseq analysis, the authors identified an interferon‐responsive macrophage (IFNRM) subset at day 3 post injury, which was reduced in aged muscle after injury. IFNRMs specifically expressed CXCL10 which promoted MuSC proliferation. Importantly, CXCL10 treatment restored muscle regeneration in aged mice (179). Another study showed that the expression of osteopontin was significantly increased in the MuSC niche in aged skeletal muscle, which suppressed the myogenic capacity of MuSCs (180). Neutralization of osteopontin improved the regeneration of aged injured muscle (180). Notably, the *Spp1* gene, which encodes osteopontin, is highly expressed by skeletal muscle macrophages in old mice (173, 180), indicating an increased pro-fibrotic activation of macrophages in aged muscle. Taken together, these findings strongly suggest that aging-related changes in infiltrating macrophages may account, in part, for the compromised regeneration of aged muscle following acute injury. Targeting these changes and rejuvenating macrophages may represent useful approaches to promote injury repair in aged muscle.

## Conclusions

It has been increasingly recognized that macrophages can acquire highly diverse functional phenotypes and play multifaceted role in skeletal muscle homeostasis, injury, and repair. They display a high level of plasticity in response to muscle microenvironment changes. In young adult skeletal muscle, macrophages contribute to the maintenance of homeostasis in the steady state. Following acute injury, infiltrating macrophages respond to the changes in injured muscle, undergoing phenotypic switch in a spatiotemporal manner to support MuSC-mediated muscle regeneration. Resident macrophages are also required for efficient clearance of necrotic fibers to promote injury repair. In chronic conditions, such as critical VML, muscular dystrophy, and aging, macrophages undergo aberrant activation to contribute to or drive muscle pathology. The aberrant activation appears mainly triggered by altered muscle microenvironment. The trained immunity that affects myeloid progenitors during hematopoiesis may also contribute. The aged immune system may contribute, in part, to the functional changes of resident macrophages, sarcopenia, and compromised acute injury repair in aged muscle. To date, our knowledge of the origins, functions, and activation mechanisms of skeletal muscle macrophages is still limited. Future studies with state-of-the-art technologies, such as lineage tracing, single cell-based high-throughput analysis, and spatial transcriptomics, will generate new valuable insights which may lead to new therapy development.

## Author contributions

LZ: Writing – review & editing. XW: Writing – original draft, Writing – review & editing.
